# TREM1 Regulates Neuroinflammatory Injury by Modulate Proinflammatory Subtype Transition of Microglia and Formation of Neutrophil Extracellular Traps *via* Interaction With SYK in Experimental Subarachnoid Hemorrhage

**DOI:** 10.3389/fimmu.2021.766178

**Published:** 2021-10-13

**Authors:** Xinyan Wu, Hanhai Zeng, Chaoran Xu, Huaijun Chen, Linfeng Fan, Hang Zhou, Qian Yu, Xiongjie Fu, Yucong Peng, Feng Yan, Xiaobo Yu, Gao Chen

**Affiliations:** Department of Neurological Surgery, The Second Affiliated Hospital, Zhejiang University School of Medicine, Hangzhou, China

**Keywords:** subarachnoid hemorrhage, TREM1, microglia, neutrophil extracellular traps, neuroinflammation

## Abstract

Neuroinflammation is a key process in the pathogenesis of subarachnoid hemorrhage (SAH) and contributes to poor outcome in patients. The purpose of this study is to explore the effect of triggering receptor expressed on myeloid cells 1 (TREM1) in the SAH, as well as its potential mechanism. In our study, plasma levels of soluble TREM1 was increased significantly after SAH and correlated to SAH severity and serum C-reactiveprotein. TREM1 inhibitory peptide LP17 alleviated the neurological deficits, attenuated brain water content, and reduced neuronal damage after SAH. Meanwhile, TREM1 inhibitory peptide decreased neuroinflammation (evidenced by the decreased levels of markers including IL-6, IL-1β, TNF-α) by attenuating proinflammatory subtype transition of microglia (evidenced by the decreased levels of markers including CD68, CD16, CD86) and decreasing the formation of neutrophil extracellular traps (evidenced by the decreased levels of markers including CitH3, MPO, and NE). Further mechanistic study identified that TREM1 can activate downstream proinflammatory pathways through interacting with spleen tyrosine kinase (SYK). In conclusion, inhibition of TREM1 alleviates neuroinflammation by attenuating proinflammatory subtype transition of microglia and decreasing the formation of neutrophil extracellular traps through interacting with SYK after SAH. TREM1 may be a a promising therapeutic target for SAH.

## Introduction

Subarachnoid hemorrhage (SAH) is a major cause of devastating outcomes after neurosurgery practice, leading to severe neurological deficits for survivors (1, 2). Early brain injury (EBI) has been thought to be a major factor affecting the prognosis after SAH (3, 4). Neuroinflammation has been considered to be a vital role in EBI (5, 6). Although great efforts that have demonstrated that inhibition of neuroinflammation could reduce neuronal damage and improve the prognosis in experimental SAH, the prognosis of SAH patients is still unsatisfactory. Accordingly, it is important to develop novel therapeutic strategies for SAH with the possibility of clinical application.

The inflammatory response of the brain to SAH is multifactorial, characterized by the innate immune system with activation of resident immune cells of the central nervous system (CNS) and a rapid infiltration of peripheral immune cells into the brain, both of which mediate inflammatory processes by multiple cytokines and chemokines (7–9). However, uncontrolled or excessive neuroinflammation is potentially harmful, leading to neuronal damage, brain edema and neurological disability in SAH (7, 10). Microglia, originating from the yolk sac, represent the major representatives of the innate immune system in the brain (11). Generally, microglia keep in a resting state. Once activated by related stimulators, they can be shifted into proinflammatory phenotype or antiinflammatory phenotype with distinct physiological functions (12–14). Microglia with proinflammatory phenotype, can detect and respond to invading pathogens through secreting proinflammatory cytokines and chemokines (including IL-6, IL-1β, TNF-α), ultimately magnifying the immune response and resulting in the exacerbation of brain damage (15, 16). These suggests that successful inhibition of neuroinflammation by suppressing the proinflammatory phenotype microglia could be an promising therapeutic strategy for SAH management. Neutrophils are innate immune cells and play a key role in immune defense and disease pathology (17). Following the onset of SAH, neutrophils infiltrate into the brain parenchyma (18, 19). Neutrophil extracellular traps (NETs), a relatively well-described pathway of neutrophil induced injury, can cause tissue-damaging immunopathology in an inflammatory state (20). Our previous articles also confirmed the role of NETs in SAH and the role of NETs in the induction of proinflammatory subtypes of microglia (17, 19). These suggests that inhibition of neuroinflammation by suppressing the formation of NETs might also be an potential therapeutic strategy for SAH management as well.

Triggering receptor expressed on myeloid cells 1 (TREM1), expressed on the surface of myeloid cells, termed the inflammation amplifier, which has a significant role in innate and adaptive immunity (21, 22). Soluble TREM1 (sTREM1) in cerebrospinal fluid of SAH patients was increased and were negatively correlated with Glasgow Coma Scale and positively correlated with the Hunt and Hess scale, suggesting that TREM1 may play a significant role in the inflammatory processes after SAH (23). TREM1 has been implicated in multiple pathological conditions including stroke, myocardial infarction, and inflammatory bowel diseases (21, 24, 25). TREM1 can recruit spleen tyrosine kinase (SYK) and interacting with SYK, which could launch downstream pro-inflammatory pathways after stroke, and inhibition of TREM1 could exert neuroprotective effects in stroke (22, 25). Previous researches also showed that SYK play an important role in inflammation and microglia polarization, which were at least partially dependent on NF-kB signaling (26, 27). In addition, studies showed that TREM1 increased the production of NETs (28, 29), and SYK participate in NETs formation (30, 31). However, the further specific mechanism of TREM1 on microglia polarization and NETs formation after SAH needs to be further identified.

Accordingly, we hypothesized that SYK-related microglia polarization and NETs formation might function downstream of TREM1 signal in SAH. However, the critical role of TREM1 in neuroinflammation following SAH remains to be elucidated. Thus, we examined whether TREM1 could mediate neuroinflammation in EBI following SAH and whether this involvement is through microglia polarization and NETs formation *via* interacting with SYK.

## Material and Method

### Statement of Ethics

This study was approved by the Ethics Committee of the Second Affiliated Hospital, Zhejiang University School of Medicine and was conducted in accordance with the principles of Good Clinical Practice and the Declaration of Helsinki. All of the patients in this study provided signed informed consent. The current study involving animals was in accordance with the Guide for the Care and Use of Laboratory Animals published by the National Institutes of Health. All protocols of this study were approved and supervised by the Institutional Animal Care and Use Committee of Zhejiang University.

### Patient Specimens

Blood was obtained from consecutive adult patients with aneurysmal SAH (within 24 h) or healthy controls in the Second Affiliated Hospital, Zhejiang University School of Medicine. Briefly, venous blood (5 mL) was collected at room temperature in 0.9% sodium citrate and centrifuged (3000g, 20 min). Liquid nitrogen was used to froze aliquots of plasma, then the frozen sample was stored at -80°C until use (32). Specimens were collected without regard for age, race, sex, or socioeconomic status.

### Animal Models of SAH

Adult male C57BL/6 mice (approximately 8 weeks) weighing 22–25 g, purchased from SLAC Laboratory Animal Company (Shanghai, China), were used to establish SAH by endovascular perforation according to the previous study (33). Briefly, after exposing the left carotid artery and its branches, a 5-0 sharpened monofilament nylon suture was advanced and finally reached the bifurcation of the anterior and middle cerebral artery. Then, vessel perforation was executed to produce SAH. The same surgical procedure without vascular perforation was implemented in the sham group.

### Animal Experimental Group Design

(**Supplementary Table S1**)

#### Experiment 1

Mice were randomly assigned to two groups including the sham group and 24 h after SAH group for quantitative real-time polymerase chain reaction (qRT-PCR).

#### Experiment 2

Mice were randomly assigned into three groups, including the sham, SAH+vehicle, and SAH+LP17 groups, for the assessment of SAH grading score, neurological function, brain water content (BWC), immunofluorescence (IF) staining, western blotting, qRT-PC, and enzyme-linked immunosorbent assay (ELISA).

#### Experiment 3

Mice were randomly assigned into two groups, including the sham and 24 h after SAH groups, for the assessment of co-immunoprecipitation (Co-IP) and double-labeled fluorescent staining.

#### Experiment 4

Mice were randomly assigned into three groups, the sham, SAH+vehicle, and SAH+Piceatannol (PIC) groups, for the assessment of SAH grading score, neurological function, western blotting, and qRT-PC.

#### Experiment 5

Mice were randomly assigned into three groups, including the SAH+vehicle, SAH+Recombinant TREM1 (rTREM1), and SAH+rTREM1+PIC groups, for the assessment of SAH grading score, neurological function, BWC, and ELISA.

For all animal samples, regions of interest are labeled in **Supplementary Figure S1**.

### Drug Administration

As previously reported, TREM1 inhibitory peptide LP17 (LQVTDSGLYRCVIYHPP) was chemically synthesized from GenScript, China (9, 25). To inhibit TREM1, vehicle or LP17 (1 mg/kg) dissolved in ultrapure water was administered intranasally 1 h post-modeling (9). Recombinant TREM-1 (GenScript, China) was intranasally administered at 1 h after SAH at the dosage of 3 μg/mouse (9). To inhibit SYK, mice were injected intraperitoneally with piceatannol (20 mg/kg, Selleck, USA) 1 h post-modeling (34).

### Severity of SAH

The 18-point SAH severity grading system was used to evaluate the severity of SAH at 24 h after SAH. The basal cistern of the mouse brain was divided into 6 segments and each part was blindly evaluated on a scale of 0–3 according to the amount of the subarachnoid blood clot (34). And mice with SAH grade score less than 8 were excluded.

### Neurological Score Evaluation

The modified Garcia scoring system and beam balance test were blindly assessed for neurological function at 24 h after SAH (9). The total score ranged from 3 to 18 for the modified Garcia scoring system and the total score ranged from 0 to 4 for the beam balance test. Higher scores for the modified Garcia scoring system and beam balance test indicated a better neurological function.

### Brain Water Content

Mouse brains were removed at 24 h post-SAH and separated into left hemisphere, right hemisphere, cerebellum and brain stem. Each part was weighed immediately to obtain the wet weight and then dried at 105°C for 72 h to obtain the dry weight. The following formula was used to calculate the the BWC: [(wet weight − dry weight)/wet weight] ×100% (33).

### Immunofluorescence Staining

Mice were euthanized at 24 hours after surgery for IF staining, which was conducted as previously described (3, 19). Brain sections with 8-μm thickness were incubated with 5% bovine serum albumin (BSA) and 0.3% Triton X-100 for 2 h at room temperature. Brain sections were incubated overnight at 4°C with primary antibodies: anti-CD68 antibody (Abcam, ab237968), anti-CD16 antibody (Invitrogen, MA1-7633), anti-CD86 antibody (Invitrogen, MA1-10299), anti-CitH3 antibody (Abcam, ab5103), anti-MPO antibody (Abcam, ab90812), anti-NE antibody (Abcam, ab68672). Sections were incubated with secondary antibodies for 2 h at room temperature. Fluoro-Jade C (FJC) staining was performed to detect neuronal damage according to the manufacturer’s protocol (Roche Inc., Basel, Switzerland). The sections were visualized using a fluorescence microscope (Leica, Germany). ImageJ software was used to analyze the results.

### Quantitative Real-Time Polymerase Chain Reaction

The left basal cortical specimen in the face of the blood clot was collected for qRT-PCR analysis 24 h after SAH. According to the manufacturer’s protocol, total mRNA was then extracted using a TRIzolTM Plus RNA Purification Kit, and we determined the quantity of the purified RNA using UV absorbance at 260 nm. Subsequently, the total RNA from each sample was used to synthesize cDNA using the PrimeScript RT Master Kit (Takara, RR420A) according to the manufacturer’s instructions. A SYBR Premix Ex Taq™ Kit (Takara, RR036A) was used for real-time PCR. The primers used are listed (**Supplementary Table S2**). The 2^−∇∇CT^ method was used to calculate the relative mRNA level of each target gene (19).

### ELISA

Determination of sTREM1 was performed as previously described (32). According to the manufacturer’s instructions, the plasma level of sTREM-1 was analyzed using a commercial ELISA kit (Human TREM1 ELISA kit, EK0844, BOSTER, China).

Determination of IL-6, IL-1β and TNF-α was performed by ELISA as previously described (35). According to the manufacturer’s instructions of the ELISA kits (BOSTER, China), homogenates of the brain from mice were prepared for detection. And we confirmed the protein levels of each sample by BCA Protein Assay Kit (Beyotime, China). Ultimately, the levels of IL-6, IL-1β and TNF-α were showed in the form of picogram/milligrams.

### Western Blotting

Left cerebral cortex was sampled for western blotting at 24 h after SAH, the procedures of which were described previously (33). Proteins from samples were extracted and prepared by using the RIPA lysis buffer. Forty micrograms of protein was separated by SDS-PAGE and transferred to PVDF membranes. Then, the PVDF membranes were blocked in blocking solution (Beyotime, China) for 1 h at room temperature. Thereafter, the membranes was incubated overnight at 4°C with primary antibodies: anti-TREM1 antibody (Abcam, ab217161), anti-p-SYK (CST, #2715T), anti-SYK (CST, #13198T), anti-Card9 (Proteintech, 10669-1-AP), anti-p-NF-κB (CST, #2715T), anti-NF-κB (CST, #8242T), anti-PAD4 (Proteintech, 17373-1-AP), anti-MPO (Santa Cruz, sc-1612-R), mouse anti-CitH3 (Abcam, ab5103), and β-actin (Abcam, ab8226). The PVDF membranes were incubated with secondary antibodies for 1 h at room temperature. Specific signals of proteins were visualized using the ECL chemiluminescence reagent kit (Millipore, USA), and protein quantification was performed by ImageJ.

### Co-IP Detection

Co-IP was conducted as previously described (25). Samples were extracted and prepared followed by centrifugation. Protein extracts of 500 μg were incubated with 1 μg of antibody against TREM-1 or control IgG overnight at 4°C. Then, the immune complexes were linked to protein A/G-agarose beads for 4 h. The eluted proteins were loaded onto SDS-PAGE gels. And the following experiment was repeated with the western blotting, but primary antibodies: anti-TREM1 antibody (Abcam, ab217161), anti-SYK (CST, #13198T), and β-actin (Abcam, ab8226).

### Statistical Analysis

For the data with a normal distribution, significant differences among groups were analyzed using Student’s t-test (2 groups) or one-way analysis of variance (ANOVA) (≥3 groups) followed by Tukey’s *post hoc* test. For the data that failed to be normally distributed, significant differences among groups were analyzed using the Mann-Whitney U test (2 groups) or Kruskal-Wallis test (≥3 groups) followed by a Dunn-Bonferroni test for *post hoc* comparisons. Associations between variables were analyzed using Spearman correlation. Mean ± standard deviation (SD) was used to express all data. *P < 0.05* indicated statistical significance. Statistical analyses were performed using GraphPad Prism and SPSS software (Version 23.0).

## Result

### Plasma sTREM1 Correlates With SAH Severity and Inflammatory Reaction in Patients With Aneurysmal SAH

**Figure 1:**
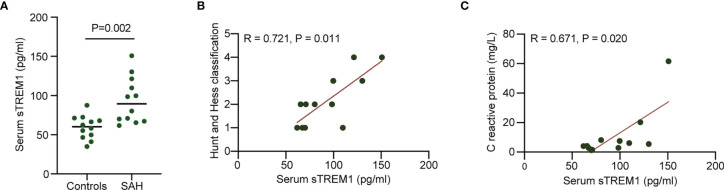
Increased plasma levels of sTREM1 in patients with aneurysmal SAH. **(A)**. The levels of plasma sTREM1 were quantified by ELISA in blood collected from control patients (n = 12) or patients with aneurysmal SAH (n = 12). P values are shown as insets. **(B**, **C)**. Correlation analysis between patient plasma sTREM1 and Hunt and Hess classification score or C reactive protein. Spearman’s rank correlation coefficient (R) and P value are shown as insets.

Plasma sTREM1 was increased in patients with patients with aneurysmal SAH (**Figure 1A**). A significant positive correlation was presented between plasma sTREM1 levels and SAH severity in patients with aneurysmal SAH (R = 0.721; P = 0.011) (**Figure 1B**). A significant positive correlation was also presented between plasma sTREM1 levels and inflammatory reaction (marked as C-reactive protein) in patients with aneurysmal SAH (R = 0.671; P = 0.020) (**Figure 1C**). These phenomena raised the possibility that not only cerebrospinal fluid (23), but plasma sTREM1 Correlates with SAH Severity and inflammatory reaction.

### Mortality, SAH Grade

The mortality in sham group was 0%. There was no significant difference in mortality among the SAH groups (**Supplementary Table S1**). When the mice were sacrificed and brain samples were collected (Sampling area showed in **Supplementary Figure S1**), no significant difference in SAH grade among the SAH groups was found (**Supplementary Figure S2**).

### Transcriptional Regulation of Inflammatory Genes in Cerebral Cortex After SAH

**Figure 2:**
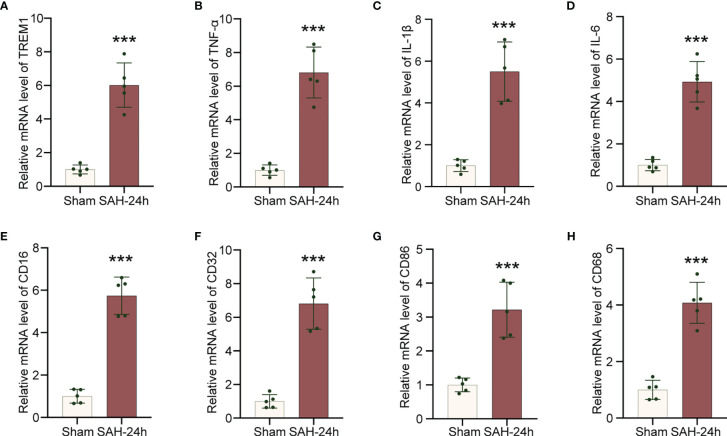
Increased levels of inflammatory genes in cerebral cortex after SAH. **(A–H)**. Real-time PCR analysis of TREM1, TNF-α, IL-1β, IL-6, CD16, CD32, CD86, and CD68. Data are expressed as mean ± SD, n = 5 in each group. ***p < 0.001 *vs.* sham group.

To explore neuroinflammatory response following SAH insult, we employed qRT-PCR to confirm that TREM1, neuroinflammation related genes (IL-6, IL-1β, TNF-α), and proinflammatory subtype transition of microglia related genes (CD68, CD16, CD32, CD86) were significantly increased in the mRNA levels following SAH (**Figures 2A–H**).

### Suppression of TREM1 Improves Neurological Function and Ameliorates Neuronal Injury

**Figure 3:**
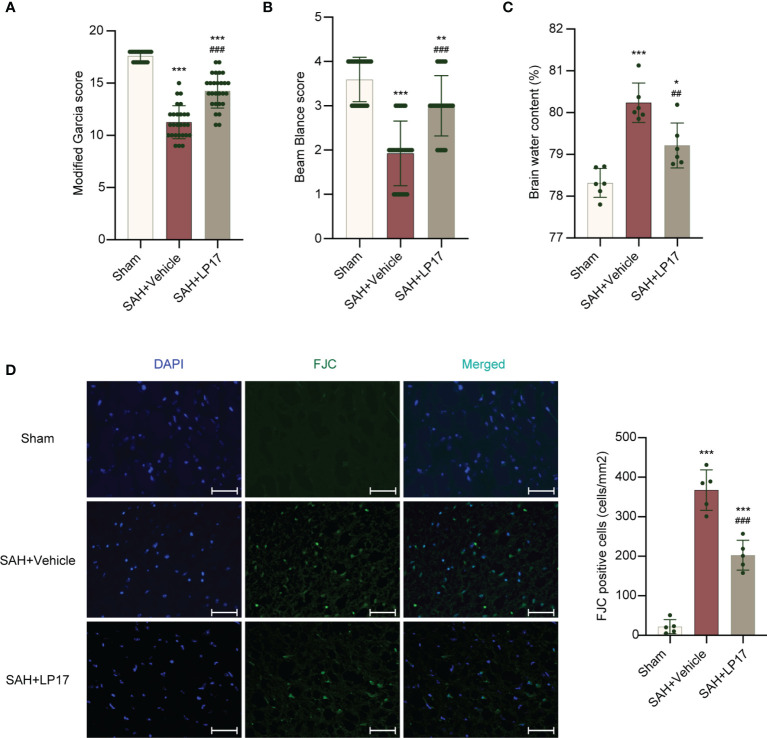
LP17 treatment improved neurological performance, attenuated brain water content, and neuron damage. **(A, B)** Quantification of neurological function. (modified Garcia score and beam balance test) at 24 h after SAH. n = 27/group. **(C)** Quantification of brain water content. n = 6/group. **(D)** Representative photograph and quantitative analysis showed the FJC positive cell (green) in different groups. n = 5/group. Data are expressed as mean ± SD. *P < 0.05, **P < 0.01, ***P < 0.001 *vs* Sham group; ^##^P < 0.01, ^###^P < 0.001 *vs* SAH+vehicle group. Scale bar = 50 μm.

We studied the effectiveness of TREM1 inhibitory peptide LP17 on neurological damage evaluated by the modified Garcia and beam balance test and brain water content at 24 h after SAH. Neurobehavioral scores and BWC results illustrated that modeling resulted in significant neurological damage when compared with sham mice (**Figures 3A–C**). However, LP17 ameliorated the neurobehavioral scores and BWC in the SAH+LP17 group compared with those in the SAH+vehicle group (**Figures 3A–C**). FJC staining also showed that LP17 inhibited the increase in the number of damage neurons after SAH (**Figure 3D**).

### Suppression of TREM1 Inhibits Proinflammatory Subtype Transition of Microglia, Formation of NETs, and Neuroinflammation

**Figure 4:**
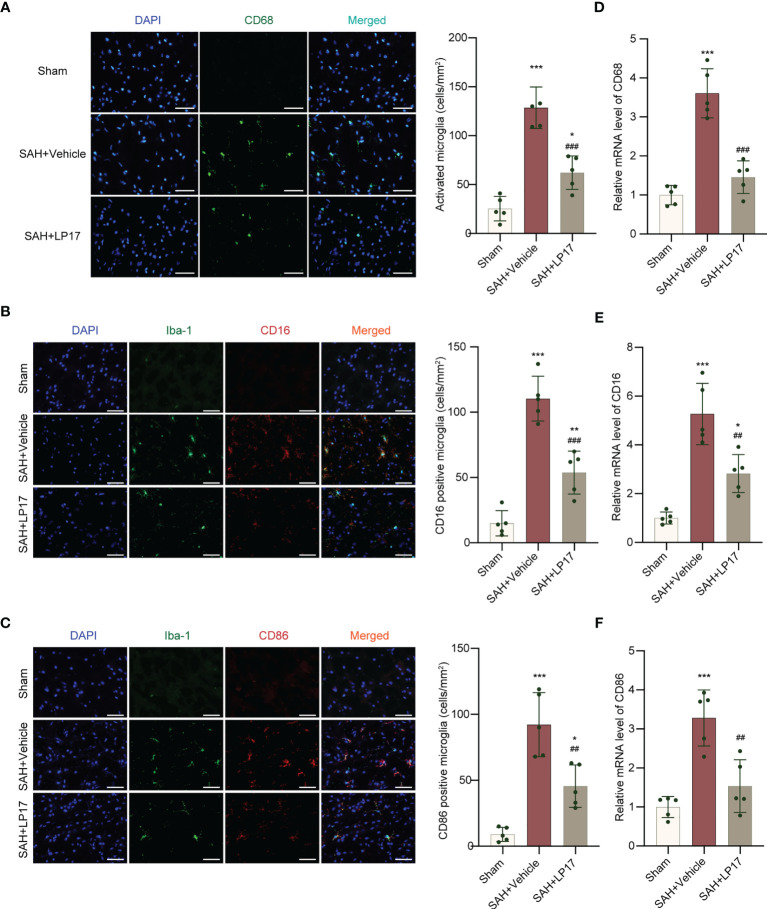
LP17 treatment inhibited activation and proinflammatory subtype transition of microglia. **(A)** Representative photograph and quantitative analysis showed the CD68 positive cell (green) in different groups. n = 5/group. **(B)** Representative photograph and quantitative analysis showed the co-localization of CD16 positive cell (red) with Iba-1 (green) in different groups. n = 5/group. **(C)** Representative photograph and quantitative analysis showed the co-localization of CD86 positive cell (red) with Iba-1 (green) in different groups. n = 5/group. **(D–F)**. Relative mRNA levels of proinflammatory subtype microglia marker genes (CD68, CD16, CD86). n = 5/group. Data are expressed as mean ± SD. *P < 0.05, **P < 0.01, ***P < 0.001 *vs* Sham group; ^##^P < 0.01, ^###^P < 0.001 *vs* SAH+vehicle group. Scale bar = 50 μm.

We visualized activated proinflammatory subtype microglia using CD68, CD16, and CD86 markers. IF analysis demonstrated that the number of CD68, CD16, and CD86 positive cells was remarkably increased in the SAH+vehicle group compared with the sham group, whereas LP17 administration decreased the the number of CD68, CD16, and CD86 positive cells (**Figures 4A–C**). Moreover, in line with IF, similar changes in the mRNA levels of CD68, CD16, and CD86 from diverse groups were also confirmed by qRT-PCR (**Figures 4D–F**).

**Figure 5:**
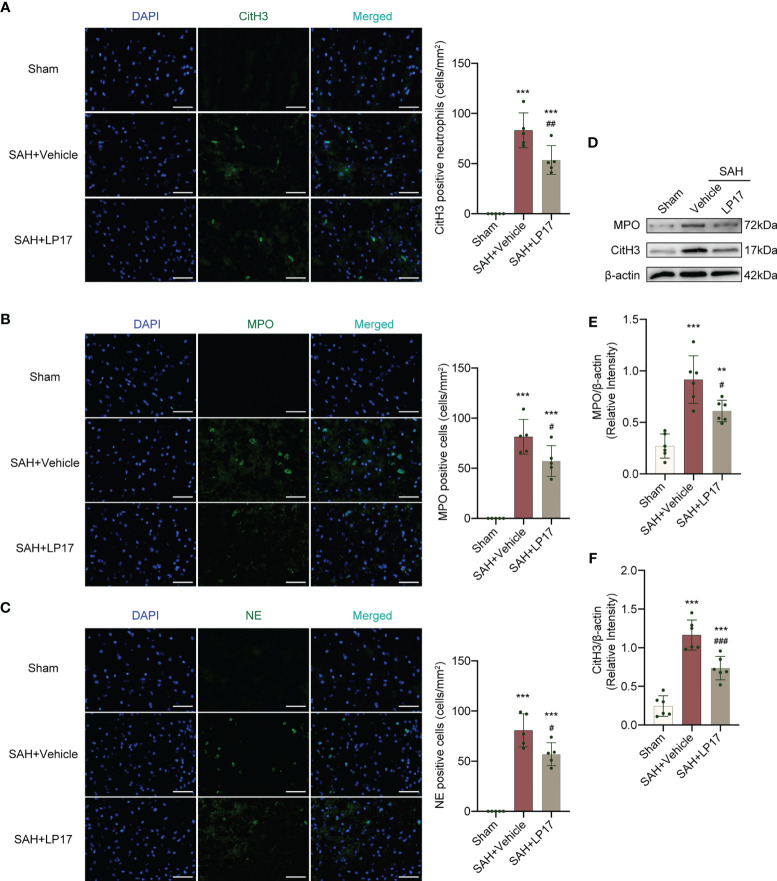
LP17 treatment inhibited the formation of NETs. **(A)** Representative photograph and quantitative analysis showed the CitH3 positive cell (green) in different groups. n = 5/group. **(B)** Representative photograph and quantitative analysis showed the MPO positive cell (green) in different groups. n = 5/group. **(C)** Representative photograph and quantitative analysis showed the NE positive cell (green) in different groups. n = 5/group. **(D–F)** Representative western blotting images and quantitative analysis of MPO and CitH3 in cortex in different groups. Control images of β-actin are re-used for illustrative purposes. n = 6/group. Data are expressed as mean ± SD. **P < 0.01, ***P < 0.001 *vs* Sham group; ^#^P < 0.05, ^##^P < 0.01, ^###^P < 0.001 *vs* SAH+vehicle group. Scale bar = 50 μm.

We visualized formation of NETs using CitH3, MPO, and NE markers. IF analysis demonstrated that the number of CitH3, MPO, and NE positive cells was remarkably increased in the SAH+vehicle group compared with the sham group, whereas LP17 administration decreased the the number of CitH3, MPO, and NE positive cells (**Figures 5A–C**). Moreover, in line with IF, similar changes in the expressions of CitH3 and MPO from diverse groups were also confirmed by western blotting (**Figures 5D–F**).

**Figure 6:**
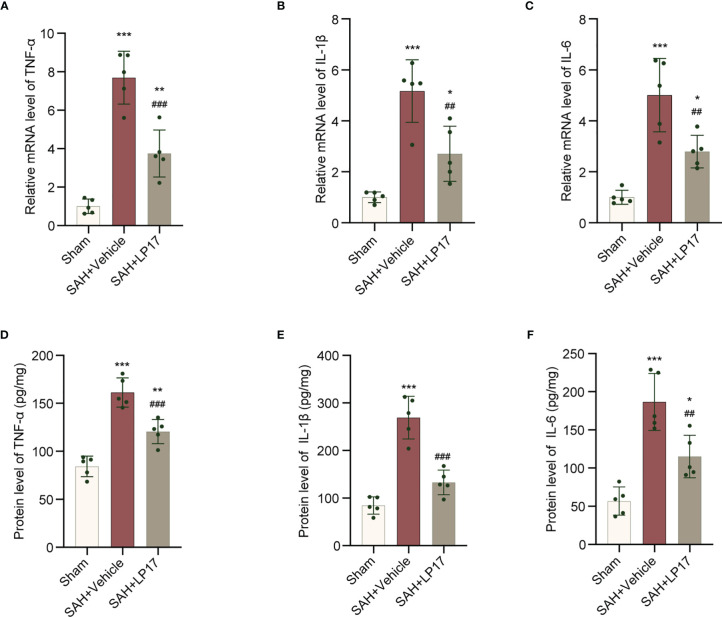
LP17 treatment inhibited neuroinflammation. **(A–C)** Relative mRNA levels of inflammatory marker genes (TNF-α, IL-1β, IL-6). n = 5/group. **(D–F)** Quantitative analysis of ELISA levels of inflammatory markers (TNF-α, IL-1β, IL-6). n = 5/group. Data are expressed as mean ± SD. *P < 0.05, **P < 0.01, ***P < 0.001 *vs* Sham group; ^##^P < 0.01, ^###^P < 0.001 *vs* SAH+vehicle group.

We visualized neuroinflammation using IL-6, IL-1β, and TNF-α markers. The data from qRT-PCR demonstrated that the levels of IL-6, IL-1β, and TNF-α was remarkably increased in the SAH+vehicle group compared with the sham group, whereas LP17 administration decreased the the levels of IL-6, IL-1β, and TNF-α (**Figures 6A–C**). Moreover, in line with qRT-PCR, similar changes in the expressions of IL-6, IL-1β, and TNF-α from diverse groups were also confirmed by ELISA (**Figures 6D–F**).

### TREM-1 Interacts With SYK

**Figure 7:**
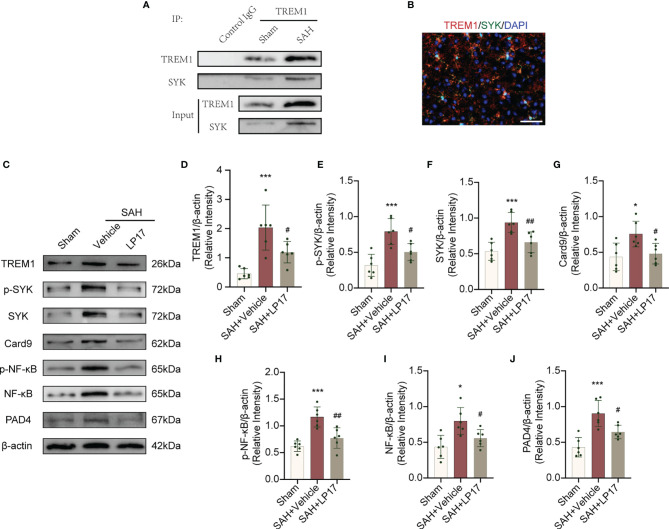
TREM1 triggered the Card9/NF-κB and PAD4-NETs signaling pathway through interacting with SYK. **(A)** The lysates from cortex tissue were immunoprecipitated with anti-TREM1. Then immunoprecipitates were analyzed by immunoblotting with anti-TREM1 and anti-SYK. n = 6/group. **(B)** Immunostaining for TREM1 and SYK in cerebral cortex. n = 3/group. Scale bar = 50 μm. **(C–J)**. Representative western blotting images and quantitative analysis of TREM1, p-SYK, SYK, Card9, p-NF-κB, NF-κB, and PAD4 in cortex in different groups. Control images of β-actin are re-used for illustrative purposes. n = 6/group. Data are expressed as mean ± SD. *P < 0.05, ***P < 0.001 *vs* Sham group; ^#^P < 0.05, ^##^P < 0.01 *vs* SAH+vehicle group.

TREM1 can recruit SYK and interacting with SYK, then leads to an activation signal to downstream components (25). We confirmed whether an association exists between TREM-1 and SYK in EBI after SAH. As showed in **Figure 7**, TREM1 presented to interact with SYK after SAH. Moreover, in line with Co-IP, co-immunolabeling further presented that TREM1 was co-localized with SYK (**Figure 7B**).

### TREM1 Is Necessary for SAH-Induced SYK Activation and Downstream Pathways

TREM1 levels, accompanied by the elevation of p-SYK and SYK, was increased after SAH (**Figures 7C–F**). SAH also induce remarkedly increased Card9, p-NF-κB, NF-κB in Card9-NF-κB pathways and PAD4 in PAD4-NETs pathways (**Figures 7C, G–J**) These increased levels of targets were all inhibited by LP17 (**Figures 7C–J**), suggesting that TREM1 could activate SYK after SAH and the activation of SYK could increase protein levels in Card9-NF-κB and PAD4-NETs signaling.

### TREM1-Induced SYK Activation Is Responsible for Proinflammatory Subtype Transition of Microglia and Formation of NETs

**Figure 8:**
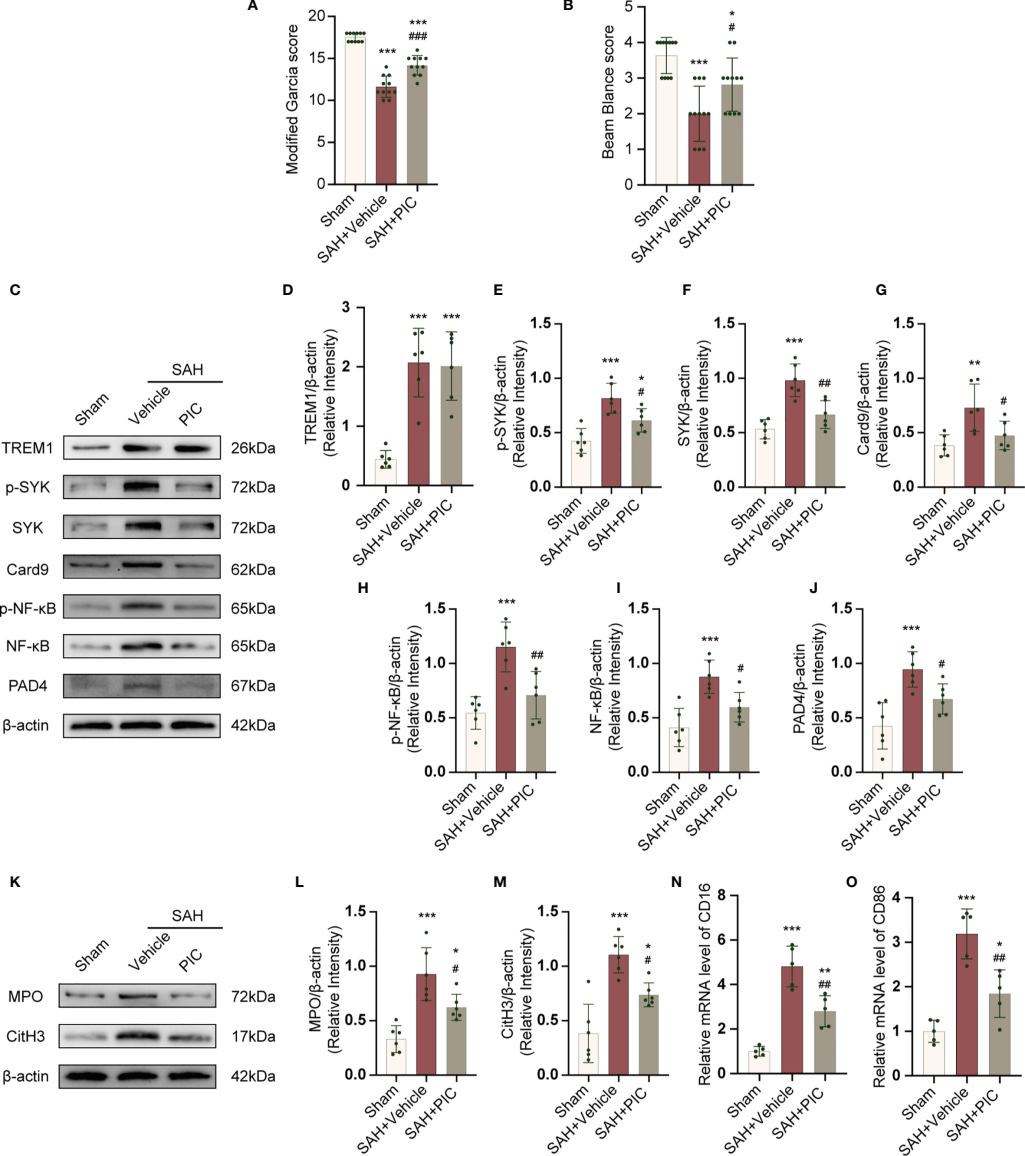
TREM1-induced SYK activation triggered proinflammatory subtype microglia and the formation of NETs after SAH. **(A, B)** Quantification of neurological function (modified Garcia score and beam balance test) at 24 h after SAH. n = 11/group. **(C–J)** Representative western blotting images and quantitative analysis of TREM1, p-SYK, SYK, Card9, p-NF-κB, NF-κB, and PAD4 in cortex in different groups. Control images of β-actin are re-used for illustrative purposes. n = 6/group. **(K–M)** Representative western blotting images and quantitative analysis of MPO and CitH3 in cortex in different groups. Control images of β-actin are re-used for illustrative purposes. n = 6/group. **(N, O)** Relative mRNA levels of proinflammatory subtype microglia marker genes (CD16, CD86). n = 5/group. Data are expressed as mean ± SD. *P < 0.05, **P < 0.01, ***P < 0.001 *vs* Sham group; ^#^P < 0.05, ^##^P < 0.01, ^###^P < 0.001 *vs* SAH+vehicle group.

PIC was used to inhibit SYK. PIC ameliorated the neurobehavioral scores (modified Garcia and beam balance test) in the SAH+PIC group compared with those in the SAH+vehicle group (**Figures 8A, B**). Without remarkedly disturbance of the increased TREM1 level, PIC injection can significantly reverse the increased levels of p-SYK, SYK, Card9, p-NF-κB, NF-κB, and PAD4 after SAH. (**Figures 8C–J**). Western blotting results showed that PIC injection can significantly reverse the increased levels of CitH3 and MPO and qRT-PCR showed that PIC injection can significantly reverse the increased levels of CD16 and CD86. (**Figures 8K–O**).

### TREM1 Triggered Neuroinflammation *via* SYK Activation

**Figure 9:**
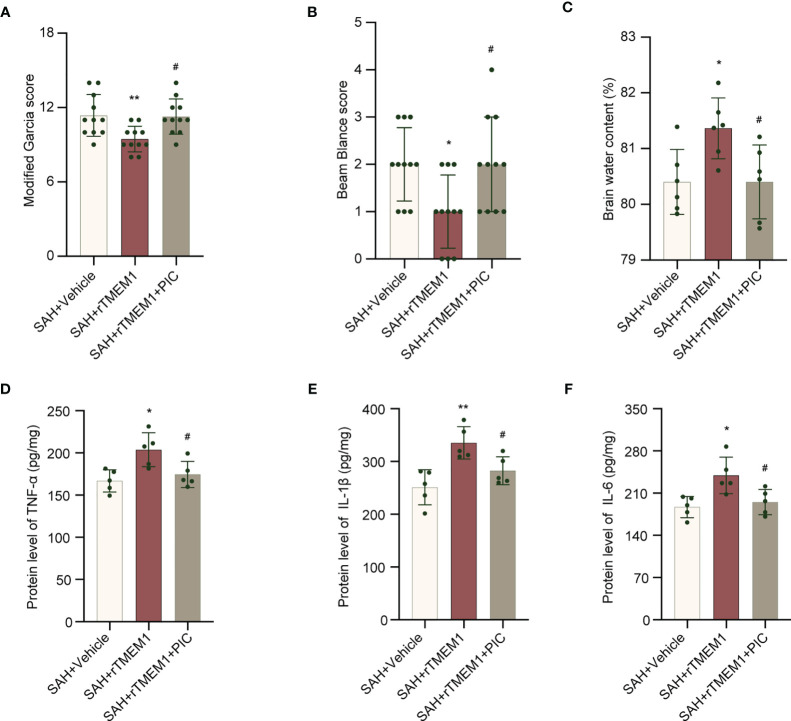
SYK inhibitor piceatannol reversed the aggravation of neurological deficits, brain edema, and inflammation induced by rTREM-1. **(A, B)** Quantification of neurological function (modified Garcia score and beam balance test) at 24 h after SAH. n = 11/group. **(C)** Quantification of brain water content. n = 6/group. **(D–F)** Relative mRNA levels of inflammatory marker genes (TNF-α, IL-1β, IL-6). n = 5/group. Data are expressed as mean ± SD. *P < 0.05, **P < 0.01 *vs* SAH+vehicle group; ^#^P < 0.05 *vs* SAH+rTREM1 group.

We treated mice with rTREM1 to increase the level of TREM1. Administration of rTREM1 aggravated the neurobehavioral scores and BWC in the SAH+rTREM1 group compared with those in the SAH+vehicle group, while administration of rTREM1 combining with PIC reversed the aggravation of neurobehavioral scores and BWC (**Figures 9A–C**). The data from ELISA also demonstrated that the levels of IL-6, IL-1β, and TNF-α was remarkably increased in the SAH+rTREM1 group compared with the SAH+vehicle, whereas administration of rTREM1 combining with PIC reversed the levels of IL-6, IL-1β, and TNF-α (**Figures 9D–F**).

## Discussion

**Figure 10:**
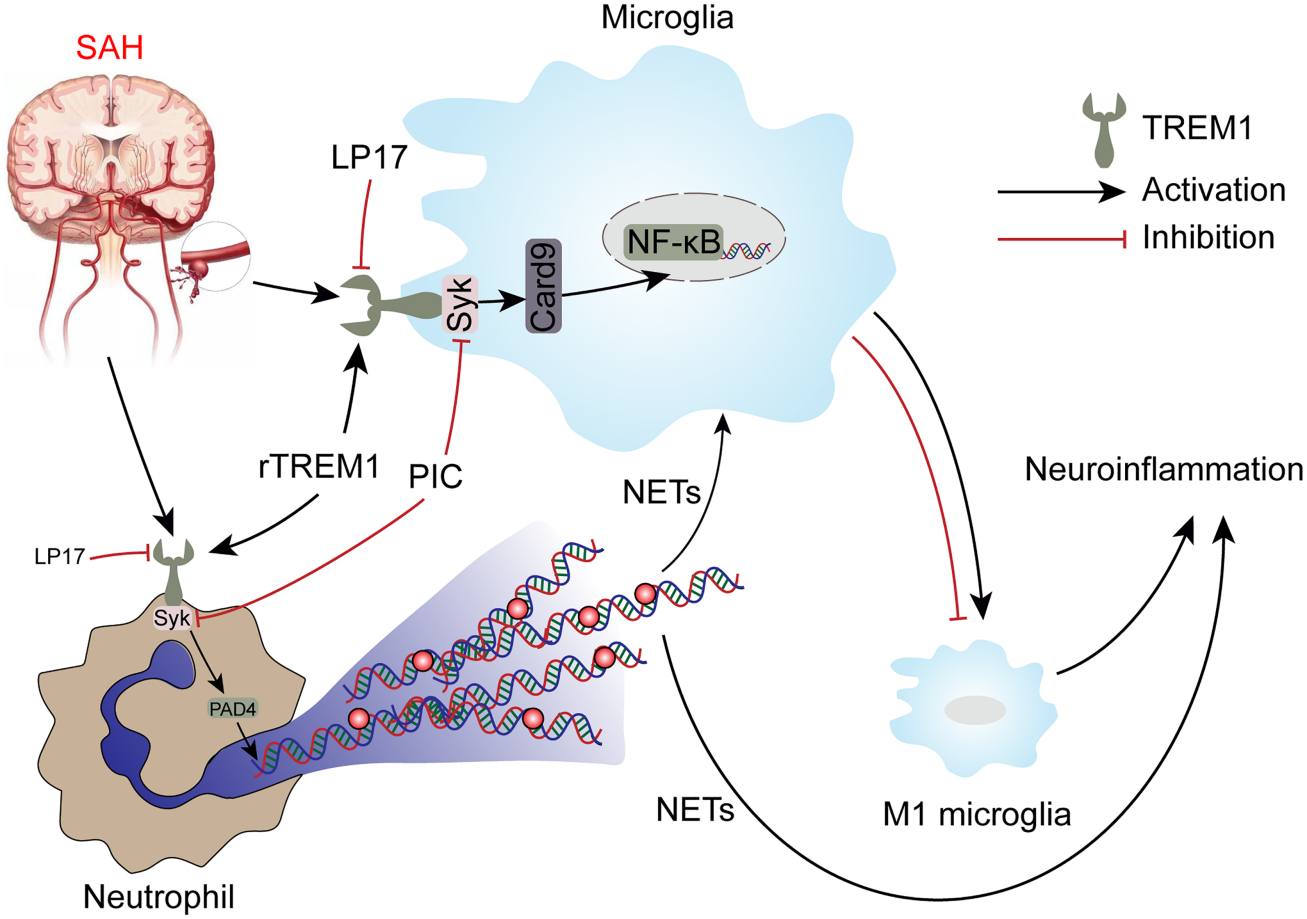
Schematic mechanism of TREM-1 regulates post-SAH neuroinflammation.

TREM1 has been reported to be a potential target for treating brain injury in CNS disease including SAH (9, 36, 37), but no study has focused on the role of TREM1 in proinflammatory subtype transition of microglia and formation of NETs following SAH. For the first time, we demonstrate the role of TREM1 in the proinflammatory subtype transition of microglia and formation of NETs following SAH and to explore the potential mechanisms. In this study, we found that TREM1 was activated after SAH in humans and mice. Pharmacological blockade of TREM1 inhibited activated proinflammatory microglia and formation of NETs, thereby relieving neuroinflammation, and thus improved neurological function. Additionally, TREM1 was demonstrated to activate SYK and interact with SYK, and inhibiting SYK abolished the effects of SAH on activated proinflammatory microglia, formation of NETs, and neuroinflammation. Moreover, TREM1 could induce neuroinflammation *via* activating SYK. Taken together, our findings indicate that TREM1 regulates neuroinflammatory injury by modulate proinflammatory subtype transition of microglia and formation of NETs *via* interaction with SYK in SAH (**Figure 10**).

After SAH, the inflammatory response of the brain mainly occur in cortical regions (27), which is also the main focus of this study. Microglia and neutrophils are key cells to the inflammatory response from CNS and peripheral immune system in SAH (27, 38). In accordance with the previous reports, our study also confirmed that animals exhibited remarkable inflammatory response in SAH (19, 27, 38). Microglia polarization and neutrophil infiltration have been proven to be the important mechanisms in the pathological process of SAH in multiple research centers including ours (9, 14, 19, 39–41). However, the upstream pathway of microglia polarization, the deep mechanism of neutrophil infiltration, and the relationship between peripheral and central immunity are relatively limited, which are also the topics that we want to explore in this study.

TREM1 was first detected in peripheral blood neutrophils and monocytes, and subsequently was detected in macrophages, endothelial cells, microglia (9, 25, 42). TREM1 is an inflammation amplifier that triggers the release of mutiple chemokines and cytokines, and induces inflammatory cascade (43, 44). TREM1 was recognized as the key role in the inflammatory response after stroke (25, 44). Numerous researches have also indicated that TREM1 inhibitors could confer protective effect in pathological conditions with excessive immune responses, including stroke, myocardial infarction, inflammatory bowel diseases, atherosclerosis, fibrosis and cancer (9, 22, 25, 28, 36, 37, 44–46). TREM1-related macrophage polarization or microglia polarization has been elucidated in recent studies (25, 47). Moreover, TREM1-related NETs formation was found in sepsis and intestinal inflammation (28, 29). However, it is the first time to reported them in SAH studies. Besides, our previous articles also confirmed the role of NETs in SAH and the role of NETs in the induction of proinflammatory subtypes of microglia (17, 19). In this study, we observed that TREM1 was upregulated after SAH and promote inflammation in cerebral cortex. TREM1 inhibitor attenuated activated proinflammatory microglia, NETs formation, and neuroinflammation. These indicate that TREM1 may contribute to neuroinflammation post-SAH.

The second major observation in this study was that TREM1 can activate Card9-NF-κB and PAD4-NETs pathways through interacting with SYK. This study found TREM1 was co-precipitated and colocalized with SYK. In terms of mechanism, TREM1 can couple with an adaptor molecule DNAX activating protein 12 kDa (DAP12) that contains an immunoreceptor tyrosine-based activation motif (ITAM) through its charged lysine residue, subsequently recruiting and mobilizing SYK (48). It has been studied that SYK can active Card9-NF-κB signaling and activating PAD4-NETs signaling through SYK-dependent reactive oxygen species production (25, 31). NF-κB plays a crucial role in transcriptional activation of many proinflammatory genes, including the genes of cytokines and chemokines (25, 49, 50). NETs itself could cause inflammation, as well as through promoting a proinflammatory subtype transition of microglia or macrophage (17, 19, 51, 52). Our results also confirmed that TREM1 inhibitory peptide LP17 relieve neuroinflammation through proinflammatory subtype transition of microglia *via* Card9-NF-κB signaling and PAD4-NETs signaling after SAH.

LP17 is an approved chemically synthesized TREM1 inhibitory peptide containing the complementary determining region-3 and the “F” β-strand of the extracellular region of TREM-1 (25). In current study, LP17 was administrated intranasally, a noninvasive delivery method that bypasses the BBB and allows direct access of therapeutic substances to the CNS. The expression of TREM1 and its association with SYK were substantially eliminated by LP17. The same was true for inflammatory expression induced by Card9-NF-κB and PAD4-NETs signaling. LP17 not only inhibited microglial inflammation associated damage, but also suppressed the crosstalk between peripheral and central immunity. However, TREM1 may also interact with other proteins in the SAH-induced immune response. Further studies are needed to obtain more details on other possible associations mediated by TREM1 in EBI after SAH.

The research of central immunity and peripheral immunity in central nervous system diseases has lasted for many years, and the interaction between them has not been well solved. This study is dedicated to solving the crosstalk between microglia and neutrophils in SAH. Although it is only a preliminary exploration, as a continuation of the research on NETs, I believe that it will have beneficial effects on neuroimmunity and clinical transformation value in the near future. Of course, there are also some shortcomings in this study. Firstly, the central and peripheral immune crosstalk has not been studied in depth. Secondly, how SYK affects PAD4 has not been fully explained.

## Conclusion

Our results demonstrated that TREM1 plays a critical role in neuroinflammation after SAH. We indicated that blockade of TREM1 can suppress inflammatory responses by attenuating proinflammatory subtype transition of microglia and decreasing the formation of neutrophil extracellular traps through interacting with SYK after SAH. Altogether, TREM1 may be a potential therapeutic target for treating SAH.

## Data Availability Statement

The raw data supporting the conclusions of this article will be made available by the authors, without undue reservation.

## Ethics Statement

The studies involving human participants were reviewed and approved by Ethics Committee of the Second Affiliated Hospital, Zhejiang University School of Medicine. The patients/participants provided their written informed consent to participate in this study. The animal study was reviewed and approved by Ethics Committee of the Second Affiliated Hospital, Zhejiang University School of Medicine.

## Author Contributions

GC and XY conceived and designed the study. HHZ, XW, CX, and HC collected the samples and performed the SAH model. HHZ and XW performed ELISA and PCR. HZ, CX, LF, and YP performed the Co-IP, western blotting and immunostaining. QY and XF prepared the figures. XY and XW analyzed data. HHZ, CX, and XW prepared the manuscript draft. XW, GC and XY revised the paper. All authors contributed to the article and approved the submitted version.

## Funding

This work was supported by the National Key R&D program of China (2018YFC1312600, 2018YFC1312603), the National Natural Science Foundation of China (No. 81771246, 81971099, 81870908), TCM Science and Technology Plan of Zhejiang province (2017ZZ013), TCM Key Discipline of Zhejiang province (2017-XK-A39), and the Natural Science Foundation of Zhejiang Province (LY19H090019).

## Conflict of Interest

The authors declare that the research was conducted in the absence of any commercial or financial relationships that could be construed as a potential conflict of interest.

## Publisher’s Note

All claims expressed in this article are solely those of the authors and do not necessarily represent those of their affiliated organizations, or those of the publisher, the editors and the reviewers. Any product that may be evaluated in this article, or claim that may be made by its manufacturer, is not guaranteed or endorsed by the publisher.

## Supplementary Material

Supplementary Figure 1 Sampling area.

Supplementary Figure 2 SAH grade.

## Abbreviations

SAH, subarachnoid hemorrhage; TREM1, triggering receptor expressed on myeloid cells 1; SYK, spleen tyrosine kinase; EBI, early brain injury; CNS, central nervous system; NETs, neutrophil extracellular traps; sTREM1, soluble TREM1; qRT-PCR, quantitative real-time polymerase chain reaction; BWC, brain water content; IF, immunofluorescence; ELISA, enzyme-linked immunosorbent assay; Co-IP, co-immunoprecipitation; PIC, piceatannol; ANOVA, one-way analysis of variance; SD, standard deviation; ITAM, immunoreceptor tyrosine-based activation motif; DAP12, DNAX activating protein 12 kDa.
