# Supplementary figures and images for: TREM1 Regulates Neuroinflammatory Injury by Modulate Proinflammatory Subtype Transition of Microglia and Formation of Neutrophil Extracellular Traps via Interaction With SYK in Experimental Subarachnoid Hemorrhage
Source: Front Immunol. 2021 Oct 13;12:766178. doi: 10.3389/fimmu.2021.766178 (PMC8548669; doi:10.3389/fimmu.2021.766178)

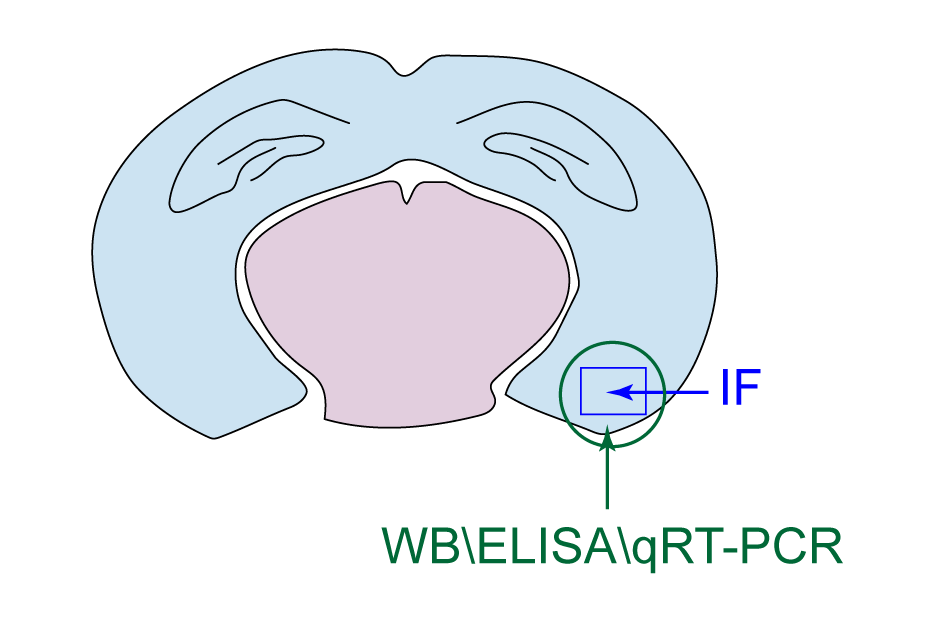

Supplement: Supplementary Figure 1 — Sampling area. [file Image_1.tif]

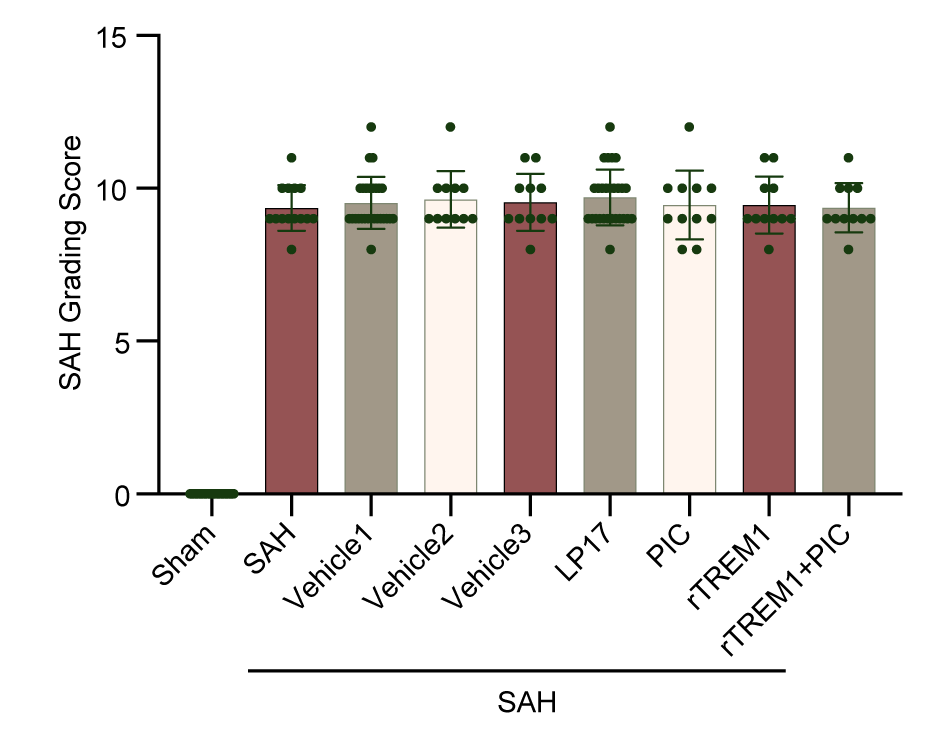

Supplement: Supplementary Figure 2 — SAH grade. [file Image_2.tif]
