# Supplementary material for: TREM1 Regulates Neuroinflammatory Injury by Modulate Proinflammatory Subtype Transition of Microglia and Formation of Neutrophil Extracellular Traps via Interaction With SYK in Experimental Subarachnoid Hemorrhage
Source: Front Immunol. 2021 Oct 13;12:766178. doi: 10.3389/fimmu.2021.766178 (PMC8548669; doi:10.3389/fimmu.2021.766178)
Supplement: Supplementary file 3 [file Table_1.docx]

Table S1, Animal experiment design, grouping, and animal mortality.

| Groups | IF | qRT-PCR | ELISA | WB | Neurological function | WBC | Mortality |
| --- | --- | --- | --- | --- | --- | --- | --- |
| **Experiment 1** |  |  |  |  |  |  |  |
| Sham |  | 5 |  |  |  |  | 0.00%(0/5) |
| SAH |  | 5 |  |  |  |  | 16.67%(1/6) |
| **Experiment 2** |  |  |  |  |  |  |  |
| Sham | 5 | 5 | 5 | 6 | 27 | 6 | 0.00%(0/27) |
| SAH+Vehicle1 | 5 | 5 | 5 | 6 | 27 | 6 | 22.86%(8/35) |
| SAH+LP17 | 5 | 5 | 5 | 6 | 27 | 6 | 18.18%(6/33) |
| **Experiment 3** |  |  |  |  |  |  |  |
| Sham | 3 |  |  | 6 |  |  | 0.00%(0/9) |
| SAH | 3 |  |  | 6 |  |  | 25.00%(3/12) |
| **Experiment 4** |  |  |  |  |  |  |  |
| Sham |  | 5 |  | 6 | 11 |  | 0.00%(0/11) |
| SAH+Vehicle2 |  | 5 |  | 6 | 11 |  | 21.43%(3/14) |
| SAH+PIC |  | 5 |  | 6 | 11 |  | 21.43%(3/14) |
| **Experiment 5** |  |  |  |  |  |  |  |
| SAH+Vehicle3 |  |  | 5 |  | 11 | 6 | 21.43%(3/14) |
| SAH+rTREM1 |  |  | 5 |  | 11 | 6 | 26.67%(4/15) |
| SAH+rTREM1+PIC |  |  | 5 |  | 11 | 6 | 21.43%(3/14) |
