# Supplementary material for: TREM1 Regulates Neuroinflammatory Injury by Modulate Proinflammatory Subtype Transition of Microglia and Formation of Neutrophil Extracellular Traps via Interaction With SYK in Experimental Subarachnoid Hemorrhage
Source: Front Immunol. 2021 Oct 13;12:766178. doi: 10.3389/fimmu.2021.766178 (PMC8548669; doi:10.3389/fimmu.2021.766178)
Supplement: Supplementary file 4 [file Table_2.docx]

**Supplementary Table S2, Primers used for qRT-PCR**

| Gene | Sense Primer (5'-3') | Antisense Primer (3'-5') |
| --- | --- | --- |
| TREM1 | ACCGCAGTGGGCTTGGGTAGGG | GAGGAAGGCTGGGCTCTGGGGACT |
| TNF-α | ATCCGCGACGTGGAACTG | ACCGCCTGGAGTTCTGGAA |
| IL-1β | TTGTTCATCTCGGAGCCTGTA | AGCACCTTCTTTTCCTTCATC |
| IL-6 | GCACTAGGTTTGCCGAGTAGA | AAGCTGGAGTCACAGAAGGAG |
| CD16 | ACTGTGGTTGGCTTTTGGGAT | GAGTGATTTCTGACTGGCTGCTG |
| CD32 | CCAGAAAGGCCAGGATCTAGTG | GGGAACCAATCTCGTAGTGTCTGT |
| CD68 | GGACTACATGGCGGTGGAAT | TGGTCACGGTTGCAAGAGAA |
| CD86 | ACGATGGACCCCAGATGCACCA | GCGTCTCCACGGAAACAGCA |
| β-Actin | GAGACCTTCAACACCCCAGC | CCACAGGATTCCATACCCAA |
